# Adsorption of Congo red on magnetic cobalt-manganese ferrite nanoparticles: Adsorption kinetic, isotherm, thermodynamics, and electrochemistry

**DOI:** 10.1371/journal.pone.0307055

**Published:** 2024-10-09

**Authors:** Xiajun Zhang, Youchao Xia, Zhou Wang

**Affiliations:** 1 The People’s Hospital of Danyang, Affiliated Danyang Hospital of Nantong University, Zhenjiang, P.R. China; 2 Zhejiang Huanke Environmental Science Research Institute Co. Ltd., Quzhou, P.R. China; 3 Vanadium and Titanium Resource Comprehensive Utilization Key Laboratory of Sichuan Province, School of Vanadium and Titanium, Panzhihua University, Panzhihua, P.R. China; SKUMS: Shahrekord University of Medical Science, ISLAMIC REPUBLIC OF IRAN

## Abstract

Magnetic Co_0.5_Mn_0.5_Fe_2_O_4_ nanoparticles were successfully prepared via the combustion and calcination process, with an average particle diameter of 31.5 nm and a saturation magnetization of 25.25 emu·g^-1^, they were employed to adsorbe Congo red (CR) from wastewater, the Pseudo-second-order kinetic and Freundlich isotherm were consistent with the adsorption data, indicating that their adsorption was a multilayer chemisorption process, the thermodynamic investigation showed that the adsorption was a favored exothermic process. The ionic strength of Cl^-^ in CR solution had no obvious effect on the adsorption efficiency of Co_0.5_Mn_0.5_Fe_2_O_4_ nanoparticles, and the maximum adsorbance was 58.3 mg·g^-1^ at pH 2, decreasing as the pH of the CR solutions increased from 2 to 12. The ion leaching experiment and XRD demonstrated that Co_0.5_Mn_0.5_Fe_2_O_4_ nanoparticles had excellent stability, and the relative removal rate was 93.85% of the first time after 7 cycles. Cyclic voltammetry and electrochemical impedance spectroscopy demonstrated that CR was adsorbed onto Co_0.5_Mn_0.5_Fe_2_O_4_ nanoparticles, and the electrical conductivity of Co_0.5_Mn_0.5_Fe_2_O_4_ nanoparticles decreased after adsorption of CR. Magnetic Co_0.5_Mn_0.5_Fe_2_O_4_ nanoparticles displayed a promising application in wastewater treatment.

## Introduction

The emergence of nanomaterials has generated a profound impact on human life. In recent years, extensive researches have been conducted on nanomaterials due to their special properties. The definition of nanomaterials is that at least one dimension of the three-dimensional space is 1–100 nm [1]. According to the properties of materials, nanomaterials can be divided into nanometer semiconductors [2], magnetic nanomaterials [3–5], nanometer superconducting materials [6], etc.

Among them, magnetic nanomaterials have a wide range of applications in the food [7], optical [8], environmental [9, 10], and biological fields [11–13] due to their advantages of magnetism and nanomaterials. In particular, magnetic nanomaterials can be used as adsorbents in the treatment of dye wastewater in the environmental field. Compared with traditional adsorbents, such as activated carbon [14], zeolite [15], activated aluminum oxide [16], etc., magnetic nanomaterials have the advantages of large specific surface area, low cost, no secondary pollution, and easy separation from wastewater under an external magnetic field [17, 18].

Spinel ferrites (MFe_2_O_4_, M = Mn, Co, Zn, Cu, Mg, etc.) are the unique magnetic nanomaterials, belonging to the cubic spinel series [19], they have been applied in many fields because of their good thermal stability, electromagnetic property, and superparamagnetic. Among them, MnFe_2_O_4_ has some characteristics, such as high saturation magnetization and low coercivity [20], and CoFe_2_O_4_ has high coercivity, good chemical stability, high electromagnetic performance, and other excellent characteristics [21], which have led to their wide applications in many fields [22–25]. The permeation of Co into MnFe_2_O_4_ could improve the coercivity and chemical stability of MnFe_2_O_4_, therefore, Co_0.5_Mn_0.5_Fe_2_O_4_ is proposed as a novel material. At present, the research and application of Co_0.5_Mn_0.5_Fe_2_O_4_ are very few, especially in the aspect of dye adsorption.

**Fig 1:**
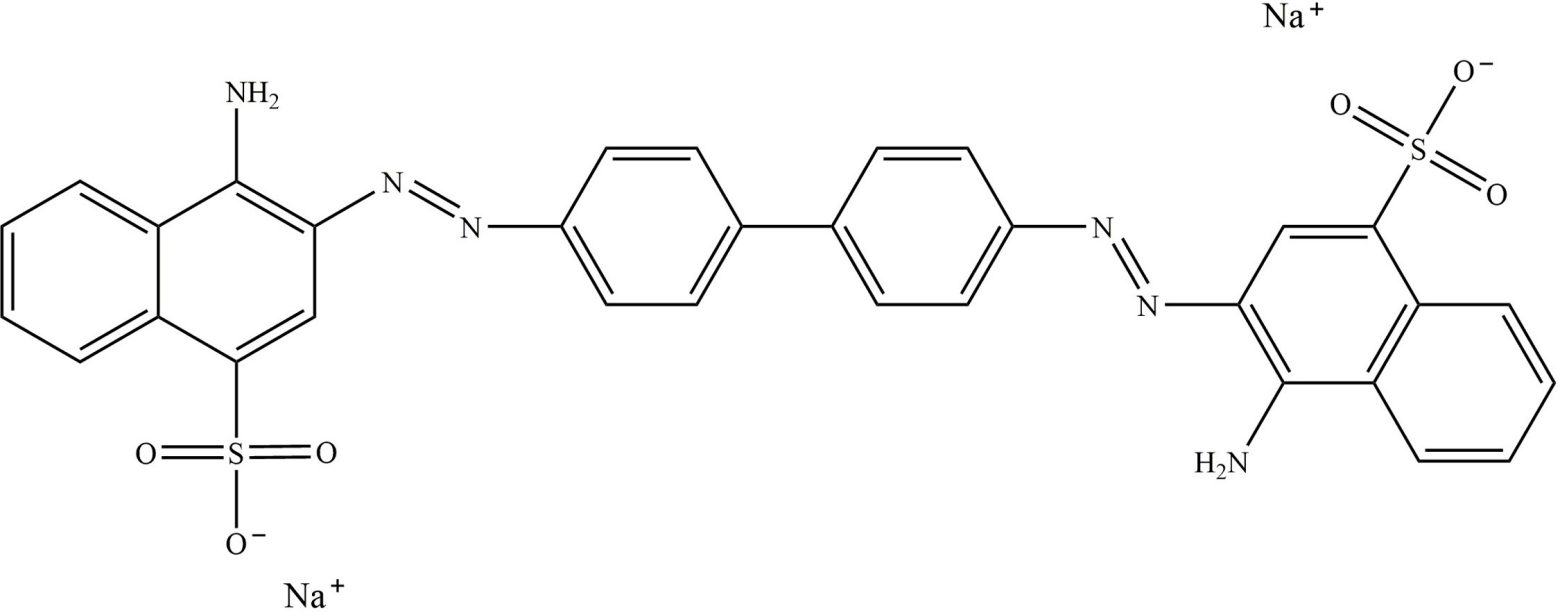
The chemical structure diagram of CR.

Congo red (CR) is a typical anionic organic dye with a complex aromatic structure [26], the molecular formula of CR is C_32_H_22_N_6_Na_2_O_6_S_2_, and the chemical structure formula is displayed in Fig 1 [27]. CR is often used in printing, textiles, food, plastic, and other industrial production [28]. It brings colorful life to humans, meanwhile, it also causes extremely serious pollution to the environment. In wastewater with different pH values, CR exists in different molecular forms which makes it different to remove CR from water, and CR is difficult to degrade due to the presence of azo groups, and its’ long-term existence in water will consume a large amount of oxygen, leading to the eutrophication of water and greatly influence human life [29], the removal of CR from wastewater becomes inevitable. As the most widely used technology in wastewater treatment, the adsorption method has the advantages such as simple operation, wide adaptability, high efficiency, stability, and no secondary pollution. However, it also has disadvantages including high cost, regeneration difficulties, and so on. Therefore, there is a need to invent a new type of adsorption material to alleviate the current drawbacks in sewage adsorption.

In this project, magnetic Co_0.5_Mn_0.5_Fe_2_O_4_ nanoparticles were prepared via the combustion and calcination process, and was employed to treat CR dye wastewater. In the adsorption studies, the effects of initial CR concentration, adsorption time, adsorption temperature, ionic strength of Cl^-^, and pH of the CR solution on the adsorption of CR by Co_0.5_Mn_0.5_Fe_2_O_4_ were investigated. Subsequently, the adsorption stability and the material recycling rate were verified.

## Materials and methods

### Materials

Manganese nitrate tetrahydrate [Mn(NO_3_)_2_∙4H_2_O, AR, 98%], cobaltous nitrate hexahydrate [Co(NO_3_)_2_∙6H_2_O, AR, 99%], ferric nitrate nonahydrate [Fe(NO_3_)_3_∙9H_2_O, AR, 98.5%] and congo red [CR, BS, 99.0%) were purchased from Sinopharm Chemical Reagent Co., Ltd (Shanghai, China), absolute ethanol (C_2_H_6_O, AR, 99.7%) was purchased from Xilong Scientific Co., Ltd. (Shantou, China).

### Preparation and characteristics of magnetic Co_0.5_Mn_0.5_Fe_2_O_4_ nanoparticles

The magnetic Co_0.5_Mn_0.5_Fe_2_O_4_ nanoparticles were prepared via the combustion and calcination process [30]. 1.10 g Mn(NO_3_)_2_·4H_2_O (98%), 1.27 g Co(NO_3_)_2_·6H_2_O (AR, 99%), and 7.05 g Fe(NO_3_)_3_·9H_2_O (AR) were added to a beaker, then 20 mL absolute ethanol (AR, 99.7%) was added rapidly and stirred in the magnetic stirring apparatus until a uniform solution formed. Then the solution was taken into the crucible, ignited, after the flame was extinguished, and the intermediate was moved into the calcinatory, and calcined at 400°C for 2 h, then the product was ground to obtain magnetic Co_0.5_Mn_0.5_Fe_2_O_4_ nanoparticles.

The morphology and composition analyses of the magnetic Co_0.5_Mn_0.5_Fe_2_O_4_ nanoparticles were investigated with scanning electron microscopy (SEM) and transmission electron microscopy (TEM), and the phase identification was analyzed by X-ray diffraction (XRD), and the corresponding composition and element proportion were analyzed by energy dispersive spectroscopy (EDS), the functional groups and chemical bonds of the nanomaterials were analyzed by Fourier Transform Infrared Spectroscopy (FTIR), and the adsorption wavelength was measured by Full-wavelength ultraviolet spectrophotometer (UV).

### Adsorption of CR onto magnetic Co_0.5_Mn_0.5_Fe_2_O_4_ nanoparticles

To investigate the adsorption mechanism of CR on magnetic Co_0.5_Mn_0.5_Fe_2_O_4_ nanoparticles and the effect of adsorption temperature on the adsorption of CR onto the magnetic Co_0.5_Mn_0.5_Fe_2_O_4_ nanoparticles. At room temperature and pH of 2, 5 mg magnetic Co_0.5_Mn_0.5_Fe_2_O_4_ nanoparticles were added to a centrifuge tube which contained 2 mL CR solution. Then the nanoparticles were dispersed for 5 min with ultrasound and stirred at different temperatures with the certain times, finally the mixtures were centrifuged, and the supernates were measured the supernatant at 497 nm [31]; NaCl was used to explore the effect of ion strength on adsorption capacity [32, 33]; the pH of CR solution required for the experiment (2, 4, 6, 8, 10, 12) was adjusted by 1 M HCl and NaOH to research the influence of pH on adsorption capacity [34]; NaSCN solution (0.1 M) was used to research whether magnetic Co_0.5_Mn_0.5_Fe_2_O_4_ nanoparticles had ion leaching phenomenon in the process of adsorbing CR; while magnetic Co_0.5_Mn_0.5_Fe_2_O_4_ nanoparticles were put into the calciner for recalcination after adsorption of CR. Then magnetic Co_0.5_Mn_0.5_Fe_2_O_4_ nanoparticles were added again into the CR solution, and the steps were repeated to study the relationship between the recycling number and adsorption capacity. The adsorption capacity of CR onto magnetic Co_0.5_Mn_0.5_Fe_2_O_4_ nanoparticles was calculated by Eq (1) [35].


$$q_{t}=\frac{(C_{0}-C_{t})\cdot V}{m}$$ (1)

Where *q*_*t*_ was (mg·g^-1^) the adsorption capacity at *t* time, *C*_0_ (mg·L^-1^) and *C*_*t*_ (mg·L^-1^) were the initial concentration of CR and the concentration at *t* time, respectively, *V* (L) was the volume of CR solution, and *m* (g) was the mass of magnetic Co_0.5_Mn_0.5_Fe_2_O_4_ nanoparticles.

### Electrochemical investigation

To investigate the change electrochemical properties of magnetic Co_0.5_Mn_0.5_Fe_2_O_4_ nanoparticles caused by the adsorption of CR onto them, at room temperature, 1 mg magnetic Co_0.5_Mn_0.5_Fe_2_O_4_ nanoparticles and 1 mg Co_0.5_Mn_0.5_Fe_2_O_4_/CR were added to 100 μL ultrapure water, respectively; then sonicated it to disperse evenly. 8 μL suspensions severally contained magnetic Co_0.5_Mn_0.5_Fe_2_O_4_ nanoparticles and Co_0.5_Mn_0.5_Fe_2_O_4_/CR were dropped onto the corresponding magnetic glassy carbon electrodes that had been milled, respectively, then dried. The working electrode was a magnetic glassy carbon electrode, the reference electrode was Ag/AgCl electrode, the counter electrode was a platinum wire electrode, and the electrolyte was 0.1 M KCl, which contained 5 mM [Fe(CN)_6_]^3-/4-^.

## Results and discussion

### Characterization of magnetic Co_0.5_Mn_0.5_Fe_2_O_4_ nanoparticles

**Fig 2:**
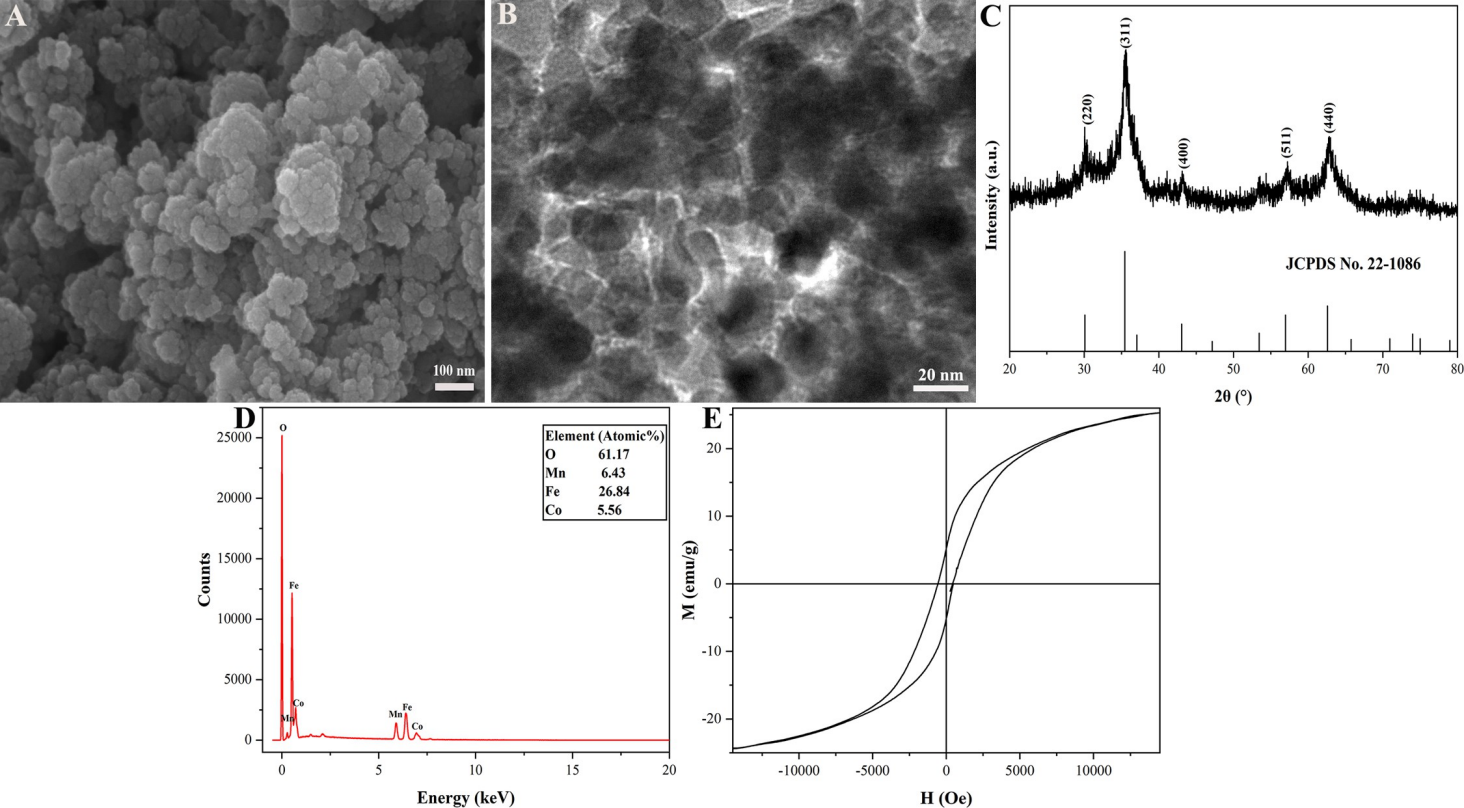
The SEM (A), TEM (B), XRD (C), EDS (D), and VSM (E) of magnetic Co_0.5_Mn_0.5_Fe_2_O_4_ nanoparticles calcined at 400°C for 2 h with 20 mL absolute ethanol.

The characteristics of magnetic Co_0.5_Mn_0.5_Fe_2_O_4_ nanoparticles were revealed in Fig 2. The SEM morphology and TEM image of magnetic Co_0.5_Mn_0.5_Fe_2_O_4_ nanoparticles were displayed in Fig 2A and 2B, respectively, magnetic Co_0.5_Mn_0.5_Fe_2_O_4_ nanoparticles were spherical and the average particle diameter was 31.5 nm. Magnetic Co_0.5_Mn_0.5_Fe_2_O_4_ nanoparticles were found to exhibit aggregation due to their high surface energy [36]. The XRD pattern of magnetic Co_0.5_Mn_0.5_Fe_2_O_4_ nanoparticles was shown in Fig 2C, the diffraction peak of magnetic Co_0.5_Mn_0.5_Fe_2_O_4_ nanoparticles was consistent with the standard PDF card (JCPDS No. 22–1086) reported by Zeng et.al [37]. The diffraction peaks of 30.08°, 35.43°, 43.05°, 56.97°, and 62.58° corresponded to (220), (311), (400), (511), and (440), respectively. The EDS spectrum of magnetic Co_0.5_Mn_0.5_Fe_2_O_4_ nanoparticles was shown in Fig 2D. The proportions of O, Mn, Fe, and Co were 61.17%, 6.43%, 26.84%, and 5.56%, respectively, which were consistent with the proportions assumed before the experiment. The hysteresis loop of magnetic Co_0.5_Mn_0.5_Fe_2_O_4_ nanoparticles was shown in Fig 2E, the saturation magnetization of the nanoparticles was 25.25 emu·g^-1^.

### Adsorption kinetics

**Fig 3:**
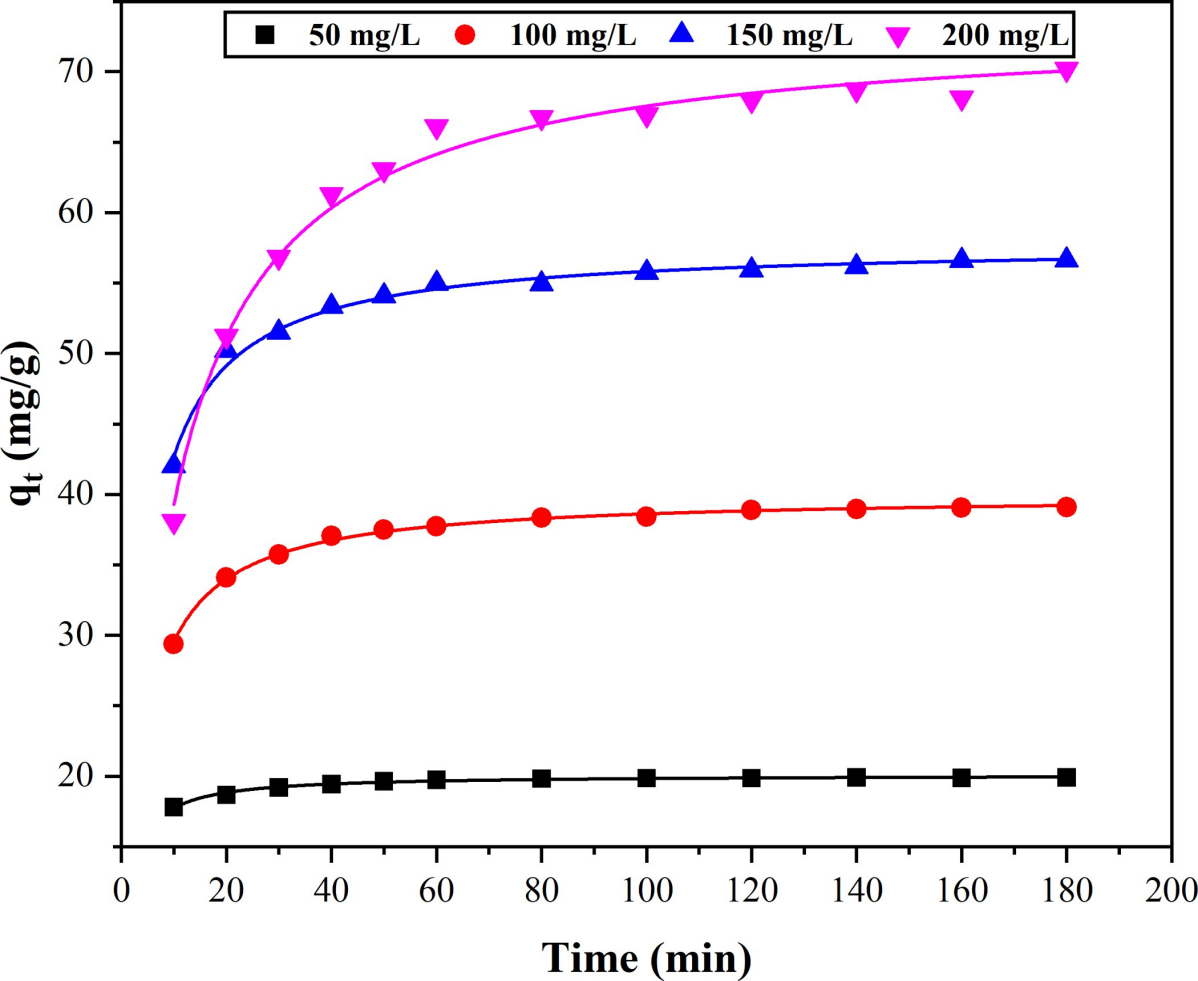
Adsorption kinetics curves of different initial CR concentrations.

The relationships between the adsorption capacity of magnetic Co_0.5_Mn_0.5_Fe_2_O_4_ nanoparticles for CR and different initial concentrations of CR were investigated. As shown in Fig 3 (the corresponding raw data were listed in S1 Table), the adsorption capacity of magnetic Co_0.5_Mn_0.5_Fe_2_O_4_ nanoparticles increased gradually with the increase of the initial CR concentration. The reason was that the adsorption sites of magnetic Co_0.5_Mn_0.5_Fe_2_O_4_ nanoparticles were much larger than what CR required. With the extension of adsorption time, the adsorption capacity of magnetic Co_0.5_Mn_0.5_Fe_2_O_4_ nanoparticles gradually increased, and the adsorption rate decreased gradually until the adsorption saturation state was reached. The reason was that the adsorption site of magnetic Co_0.5_Mn_0.5_Fe_2_O_4_ nanoparticles was gradually occupied with the increase of adsorption time, meanwhile, the functional groups on the surface of magnetic Co_0.5_Mn_0.5_Fe_2_O_4_ nanoparticles were gradually reduced [38, 39].

To study the adsorption mechanism of CR onto magnetic Co_0.5_Mn_0.5_Fe_2_O_4_ nanoparticles, the pseudo-first-order model, pseudo-second-order model, and intraparticle diffusion model were used to fit and analyze the experimental data. Pseudo-first-order model assumed that the adsorption process was physical adsorption, the pseudo-second-order model assumed that the adsorption was a chemical process [40], and the intraparticle diffusion model assumed that the adsorption rate was controlled by the internal diffusion of the adsorbent [41]. Eqs (2–4) of the pseudo-first-order model, pseudo-second-order model, and intraparticle diffusion model were as followed, respectively.


$$q_{t}=q_{e}(1-e^{-k_{1}t})$$ (2)

$$q_{t}=\frac{q_{e}^{2}k_{2}t}{1+q_{e}k_{2}t}$$ (3)

$$q_{t}=x_{i}+k_{i}t^{\frac{1}{2}}$$ (4)

Where *q*_*t*_ (mg·g^-1^) and *q*_*e*_ (mg·g^-1^) were the adsorption capacity at *t* time and at equilibrium time, respectively. *k*_1_ (min^-1^), *k*_2_ (g·mg^-1^·min^-1^), and *k*_*i*_ (mg·g^-1^·min^-0.5^) were equilibrium rate constants of three models, respectively. *x*_*i*_ was the thickness of the adsorbed boundary layer.

**Fig 4:**
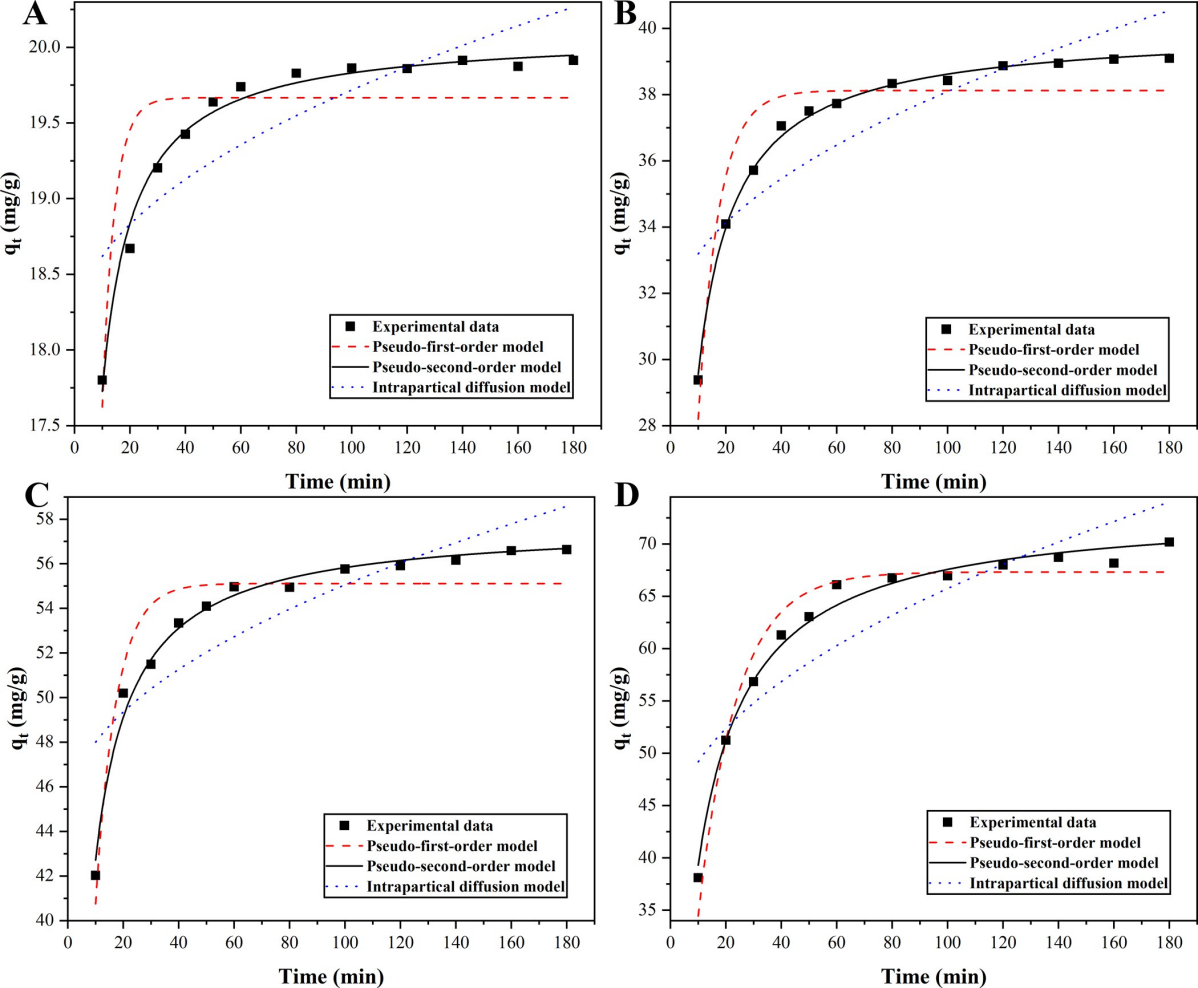
Effects of adsorption time and initial CR concentration (50 mg·L^-1^ (A), 100 mg·L^-1^ (B), 150 mg·L^-1^ (C), 200 mg·L^-1^ (D)) on the adsorption capacity of magnetic Co_0.5_Mn_0.5_Fe_2_O_4_ nanoparticles.

The kinetic fitting curves of magnetic Co_0.5_Mn_0.5_Fe_2_O_4_ nanoparticles under different initial concentrations of CR and various adsorption times were shown in Fig 4 (the corresponding raw data were listed in S1 Table). It was shown that the pseudo-second-order kinetic model best fitted the experimental data. It could be seen from Table 1 that the correlation coefficient (*R*^2^) (0.9879–0.9974) of the pseudo-second-order kinetic model was greater than those of the other two kinetic models at different initial concentrations of CR, indicating that the pseudo-second-order kinetic model could be used to describe the adsorption process of CR onto magnetic Co_0.5_Mn_0.5_Fe_2_O_4_ nanoparticles. Many researches of CR adsorption followed the pseudo-second-order kinetic model. At each initial concentration, the theoretical adsorption capacity of the equilibrium state determined by the pseudo-second-order kinetic model was close to the experimental value [39]. The results suggested that the adsorption process of CR onto magnetic Co_0.5_Mn_0.5_Fe_2_O_4_ nanoparticles was chemisorption.

**Table 1:** Fitted kinetics parameters for adsorptions of CR in aqueous solution onto magnetic Co_0.5_Mn_0.5_Fe_2_O_4_ nanoparticles at room temperature.

Kinetic models	Equations	Parameters	Initial concentration of CR (mg·L^-1^)			
			50	100	150	200
**Pseudo-first-order model**	$q_{t}=q_{e}(1-e^{-k_{1}t})$	*q*_*e*_	19.6665	38.1201	55.1119	67.3114
		*k*_*1*_	0.2264	0.1346	0.1345	0.0717
		*R* ^2^	0.7449	0.8763	0.8945	0.9559
		Adj. *R*^2^	0.7194	0.8639	0.8839	0.9515
**Pseudo-second-order model**	$q_{t}=\frac{q_{e}^{2}k_{2}t}{1+q_{e}k_{2}t}$	*q*_*e*_	20.0952	39.9808	57.7983	73.4345
		*k*_2_	0.0372	0.0071	0.0049	0.0016
		*R* ^2^	0.9879	0.9974	0.9885	0.9899
		Adj. *R*^2^	0.9867	0.9971	0.9874	0.9889
**Intraparticle diffusion model**	$q_{t}=x_{i}+k_{i}t^{\frac{1}{2}}$	*x*_*i*_	18.1113	30.9214	44.7349	41.5206
		*k*_*i*_	0.1606	0.7168	1.0318	2.4214
		*R* ^2^	0.6872	0.7074	0.6896	0.7426
		Adj. *R*^2^	0.6559	0.6782	0.6586	0.7168

### Adsorption isotherm

**Fig 5:**
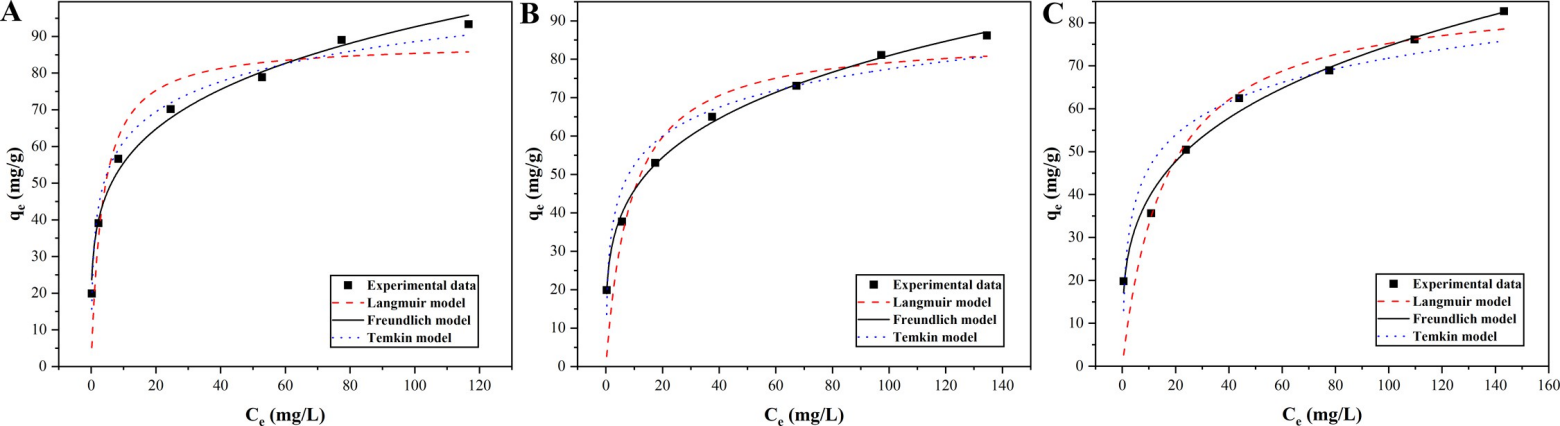
Adsorption isotherms of CR onto magnetic Co_0.5_Mn_0.5_Fe_2_O_4_ nanoparticles at 303 K (A), 313 K (B), and 323 K (C).

The relationships between the adsorption capacities of magnetic Co_0.5_Mn_0.5_Fe_2_O_4_ nanoparticles and the concentrations of CR solutions at different adsorption temperatures (303 K, 313 K, 323 K) were investigated. As shown in Fig 5 (the corresponding raw data were displayed in S2 Table), the Langmuir model, Freundlich model, and Temkin model were applied to analyze the experimental data [42, 43]. The Langmuir model assumed that the adsorption process took place on a homogeneous surface, the Freundlich model assumed that the heterogeneous surface underwent multilayer adsorption, and the Temkin model assumed that the adsorption process was a mixed process of monomolecular layer and multimolecular layer. These Eqs (5–7) were as follows:

$$q_{e}=\frac{q_{\max}K_{L}C_{e}}{1+K_{L}C_{e}}$$ (5)

$$q_{e}=K_{F}C_{e}^{\frac{1}{n}}$$ (6)

$$q_{e}=B\text{ln}\left(A_{T}C_{e}\right)$$ (7)

Where *q*_*e*_ (mg·g^-1^) and *q*_*max*_ (mg·g^-1^) were the adsorption capacity at equilibrium time and the maximum adsorption capacity of CR onto magnetic Co_0.5_Mn_0.5_Fe_2_O_4_ nanoparticles, respectively. *C*_*e*_ (mg·L^-1^) was the equilibrium concentration. *K*_*L*_ and *K*_*F*_ were the rate constants of the Langmuir model and the Freundlich model, respectively. 1/*n* was the adsorption intensity of the Freundlich model. *A*_*T*_ and *B* were constants of the Temkin model.

In Fig 5, *q*_*e*_ gradually increased with the increase of *C*_*e*_. Obviously, the Freundlich isotherm model could better fit the experimental data at different temperatures. As listed in Table 2, the *R*^2^ values of the Freundlich isotherm model (0.9851–0.9968) were higher than those of the other two isotherm models at different temperatures. The parameter *n* of the Freundlich isotherm model was used to describe the adsorption strength. 1/*n* was great than 0 and less than 1 at different temperatures, indicating that the adsorption process was favorable [44], and the adsorption mechanism of CR onto magnetic Co_0.5_Mn_0.5_Fe_2_O_4_ nanoparticles was a multilayer chemisorption process. Similarly reported adsorption of CR could also be explained by using the Freundlich isotherm model, such as rod-shaped silver nanoparticles-functioned biogenic hydroxyapatite and PVDF/*β*-CD membrane [45].

**Table 2:** The parameter values for Langmuir, Freundlich and Temkin models for CR onto magnetic Co_0.5_Mn_0.5_Fe_2_O_4_ nanoparticles at different temperatures (303 K, 313 K, 323 K).

*T*(K)	Langmuir			Freundlich			Temkin		
	*K*_*L*_	*q*_*max*_	*R* ^2^	*K*_*F*_	1/*n*	*R* ^2^	*B*	*A*_*T*_	*R* ^2^
**303**	0.2874	88.3366	0.8861	33.2204	0.2226	0.9885	11.8973	17.1156	0.9801
**313**	0.1133	86.0786	0.8637	25.9891	0.2467	0.9968	10.8682	12.4403	0.9338
**323**	0.0609	87.5694	0.8719	20.7081	0.2784	0.9851	11.1113	6.3903	0.8965

The maximum adsorption capacities of CR onto magnetic Co_0.5_Mn_0.5_Fe_2_O_4_ nanoparticles and other adsorbents were listed in Table 3, it was obvious that the maximum adsorption of magnetic Co_0.5_Mn_0.5_Fe_2_O_4_ nanoparticles had a significant advantage over other adsorbents. Although the adsorption temperature was more demanding than that of some adsorbents, was only 30°C and cannot be regarded as a disadvantage. Furthermore, the adsorption performance of magnetic Co_0.5_Mn_0.5_Fe_2_O_4_ nanoparticles increased with the initial concentration of CR, which would be favorable for future large-scale practical applications. In addition, magnetic Co_0.5_Mn_0.5_Fe_2_O_4_ nanoparticles were easy to be separated from CR solution after adsorption under an external magnetic field, suggesting that magnetic Co_0.5_Mn_0.5_Fe_2_O_4_ nanoparticles had a good application prospect.

**Table 3:** Compared the maximum adsorption capacity of CR onto magnetic Co_0.5_Mn_0.5_Fe_2_O_4_ nanoparticles with other adsorbents.

Adsorbents	Dose (mg·mL^-1^)	Temperature (^o^C)	*q*_*max*_ (mg·g^-1^)	References
Coated Fe_3_O_4_ nanoparticles	0.2	25	50	[57]
Vernonia amygdalina leaf powder	3.33	25	57.47	[58]
CCO@450	0.8	RT	59.4	[45]
CCO@550	0.8	RT	43.7	[45]
WS_400-4_	2	30	69.013	[59]
Magnetic Co_0.5_Mn_0.5_Fe_2_O_4_ nanoparticles	2.5	30	88.3366	This work

Adsorption thermodynamics

Thermodynamic studies were mainly applied to judge whether magnetic Co_0.5_Mn_0.5_Fe_2_O_4_ nanoparticles adsorbed CR favorably [46]. Thermodynamic parameters (*ΔG*^0^, *ΔS*^0^, *ΔH*^0^) were used to explain the effect of temperature on the adsorption capacity of CR onto magnetic Co_0.5_Mn_0.5_Fe_2_O_4_ nanoparticles. Thermodynamic parameters were calculated according to Eqs (8–10) [47].


$$K_{0}=\frac{q_{e}}{C_{e}}$$ (8)

$$\Delta G^{0}=-RT\text{ln}K_{0}$$ (9)

$$\text{ln}K_{0}=-\frac{\Delta H^{0}}{R}\frac{1}{T}+\frac{\Delta S^{0}}{R}$$ (10)

Where *q*_*e*_ (mg·g^-1^) was the equilibrium adsorption capacity of CR onto magnetic Co_0.5_Mn_0.5_Fe_2_O_4_ nanoparticles, *C*_*e*_ (mg·L^-1^) was the concentration of CR solution at adsorption equilibrium, *R* (8.314 J·K^-1^mol^-1^) was the gas constant, *T* (K) was kelvin temperature, *K*_0_ was the thermodynamic equilibrium constant, *ΔG*^0^ could be calculated according to Eq (9), *ΔS*^0^ and *ΔH*^0^ could be calculated according to Eq (10).

**Fig 6:**
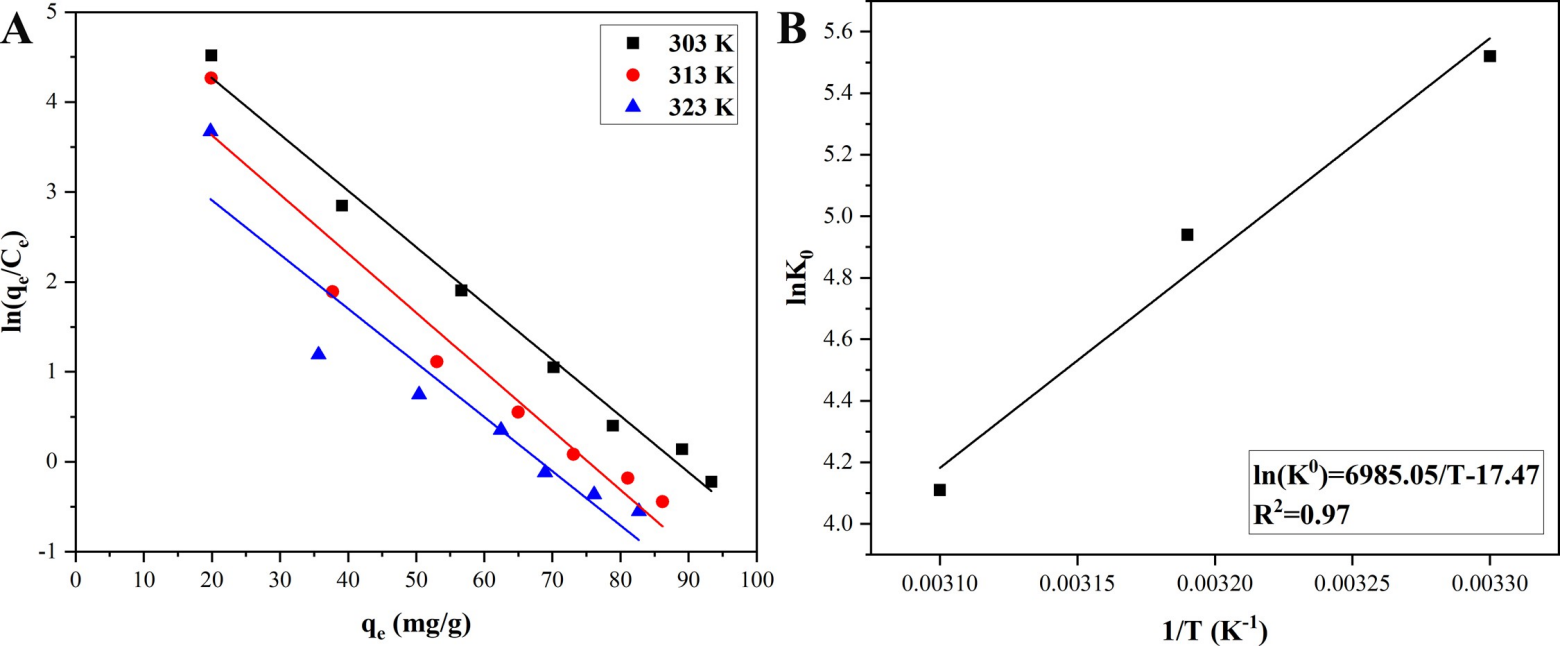
Plots of ln(q_e_/C_e_) vs. q_e_ (A) and linear van’t Hoff (B) for CR adsorbed onto magnetic Co_0.5_Mn_0.5_Fe_2_O_4_ nanoparticles at different temperatures.

The adsorption capacities of CR onto magnetic Co_0.5_Mn_0.5_Fe_2_O_4_ nanoparticles at different temperatures were shown in Fig 5, the adsorption capacities decreased gradually with the increase of the temperature. The plot of ln(*q*_*e*_/*C*_*e*_) vs. *q*_*e*_ for CR adsorbed onto magnetic Co_0.5_Mn_0.5_Fe_2_O_4_ nanoparticles at different temperatures was revealed in Fig 6A, the value of ln(*q*_*e*_/*C*_*e*_) at zero coverage was ln*K*_0_. At 303 K, 313 K, and 323 K, ln*K*_0_ values were 5.52, 4.94, and 4.11, respectively. The *R*^2^ values of their linear regressions were 0.99, 0.94, and 0.89, respectively. The plot of the linear van’t Hoff was shown in Fig 6B, and the thermodynamic parameters were listed in Table 4, ln*K*_0_ decreased with the increase of temperature and *ΔH*^0^ was -45.73 KJ·mol^-1^, the result revealed that the adsorption process of CR onto magnetic Co_0.5_Mn_0.5_Fe_2_O_4_ nanoparticles was exothermic [48]. At 303 K, 313 K, and 323 K, *ΔG*^0^ values were -13.91 KJ·mol^-1^, -12.86 KJ·mol^-1^, and -11.04 KJ·mol^-1^, respectively. *ΔG*^0^ values were less than zero at each temperature, indicating that the adsorption of CR onto magnetic Co_0.5_Mn_0.5_Fe_2_O_4_ nanoparticles was a favored process [49]. *ΔS*^0^ was less than zero, indicating that the freedom degree of CR adsorbed onto magnetic Co_0.5_Mn_0.5_Fe_2_O_4_ nanoparticles decreased [50].

**Table 4:** Thermodynamic parameters of CR adsorbed onto magnetic Co_0.5_Mn_0.5_Fe_2_O_4_ nanoparticles.

*T*/K	*ΔG*^0^/(KJ·mol^-1^)	*ΔH*^0^/(KJ·mol^-1^)	*ΔS*^0^/(J·mol^-1^·K^-1^)
**303**	-13.91	-45.73	-105.00
**313**	-12.86		
**323**	-11.04		

### Adsorption mechanism of CR onto magnetic Co_0.5_Mn_0.5_Fe_2_O_4_ nanoparticles

#### FTIR analysis

**Fig 7:**
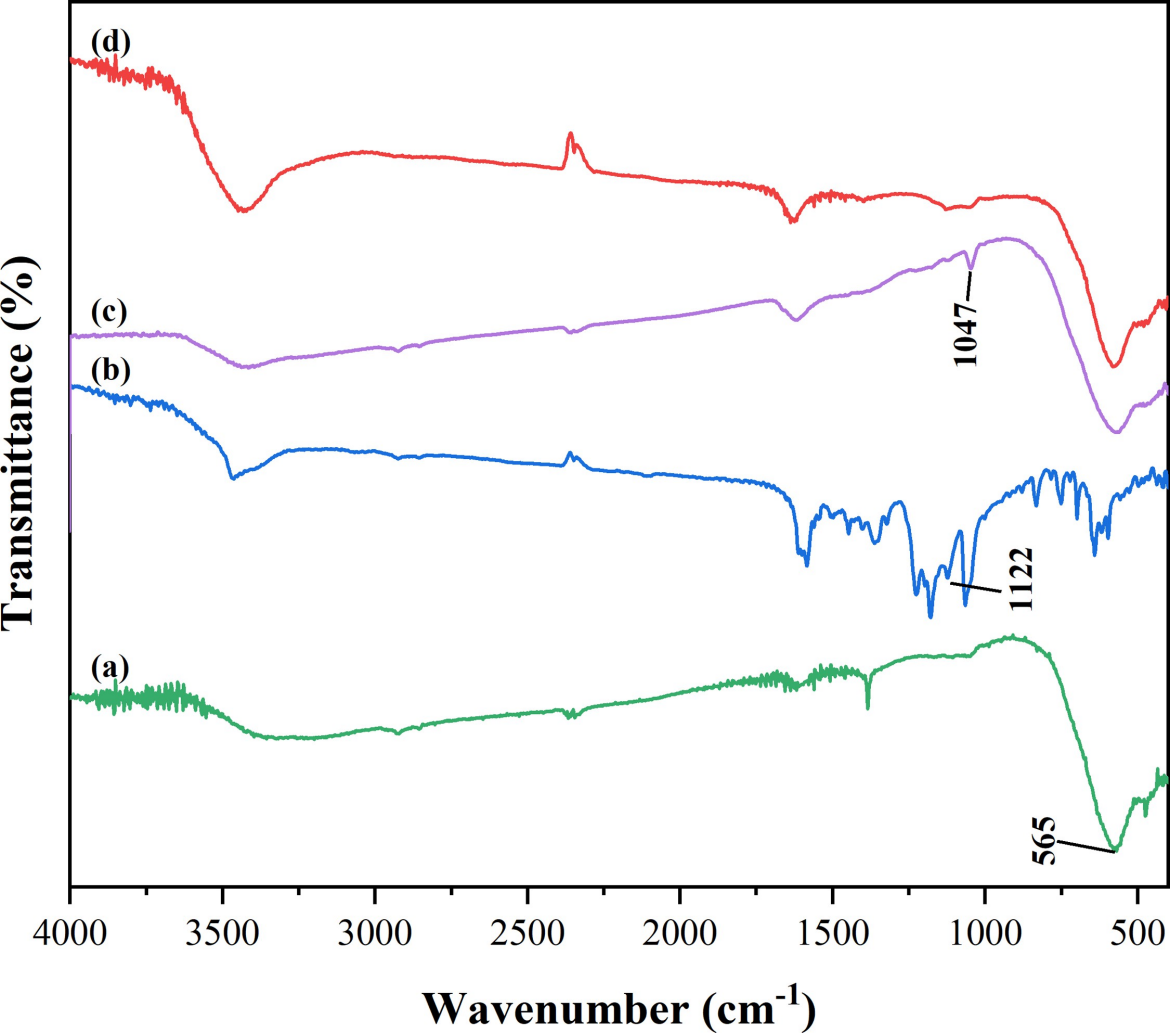
FTIR spectra of magnetic Co_0.5_Mn_0.5_Fe_2_O_4_ nanoparticles (a), CR (b), magnetic Co_0.5_Mn_0.5_Fe_2_O_4_ nanoparticles after adsorbing CR (c), magnetic Co_0.5_Mn_0.5_Fe_2_O_4_ nanoparticles after adsorbed re-calcined (d).

The FTIR spectra of magnetic Co_0.5_Mn_0.5_Fe_2_O_4_ nanoparticles, CR, magnetic Co_0.5_Mn_0.5_Fe_2_O_4_ nanoparticles after adsorbing CR, and magnetic Co_0.5_Mn_0.5_Fe_2_O_4_ nanoparticles after adsorbed re-calcined were displayed in Fig 7. As shown in Fig 7(A)–7(C), the peak at 565 cm^-1^ represented the characteristic peak of Fe-O, while the peaks at 1122cm^-1^ and 1047 cm^-1^ indicated the -SO_3_H stretching vibration of CR, revealing that CR was adsorbed onto magnetic Co_0.5_Mn_0.5_Fe_2_O_4_ nanoparticles. The -SO_3_H bond of CR shifted from 1122 cm^-1^ to 1047 cm^-1^, the reason was that it was caused by the action of chemical bonds in the adsorption process [51]. Fig 7(D) displayed that the characteristic peak of CR disappeared after the re-calcination of magnetic Co_0.5_Mn_0.5_Fe_2_O_4_ nanoparticles, indicating that the adsorption of CR onto magnetic Co_0.5_Mn_0.5_Fe_2_O_4_ nanoparticles was a chemisorption process. The result was consistent with the conclusion of the isotherm model.

#### Effects of ion strength and pH on the adsorption capacity and recycling of Co_0.5_Mn_0.5_Fe_2_O_4_ nanoparticles

**Fig 8:**
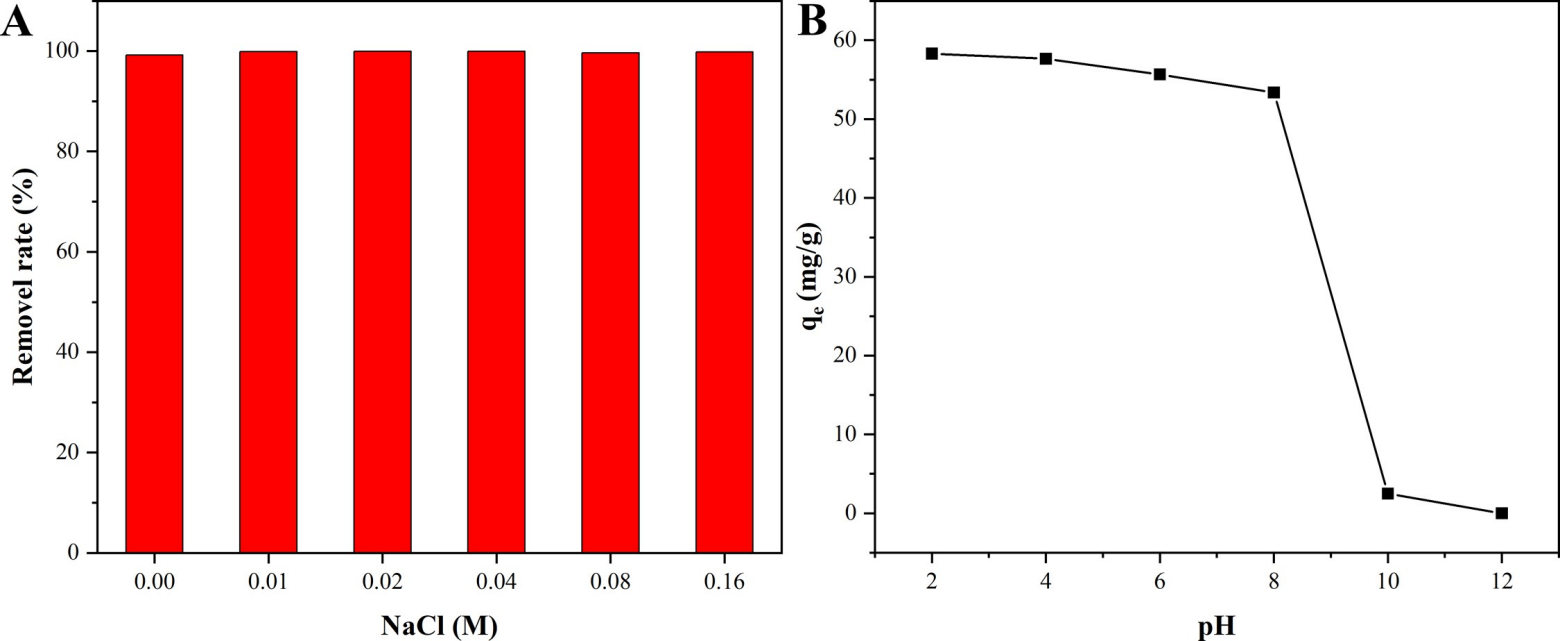
Effect of ion strength (A), and pH (B) on the equilibrium adsorption capacity of CR on magnetic Co_0.5_Mn_0.5_Fe_2_O_4_ nanoparticles.

The effect of NaCl in dye wastewater on the adsorption of CR onto magnetic Co_0.5_Mn_0.5_Fe_2_O_4_ nanoparticles was shown in Fig 8A (the corresponding raw data were revealed in S3 Table), the removal rate of CR was stable at more than 99%. The removal rate had no obvious change with the increase of NaCl ion strength, indicating that Cl^-^ did not occupy the active sites of magnetic Co_0.5_Mn_0.5_Fe_2_O_4_ nanoparticles and there was no competition with CR [52]. The change of pH affected the adsorption capacity of the adsorbent, which was mainly due to the surface ionization of the adsorbent in different pH solutions and the ionic state of the adsorbate [53]. As shown in Fig 8B (the corresponding raw data were listed in S4 Table), the adsorption capacity of CR onto magnetic Co_0.5_Mn_0.5_Fe_2_O_4_ nanoparticles decreased gradually when the pH of solution increased from 2 to 8, and the maximum adsorption capacity was 58.3 mg·g^-1^ at pH of 2. With the pH of CR solution increasing from 8 to 12, the adsorption capacity of CR onto magnetic Co_0.5_Mn_0.5_Fe_2_O_4_ nanoparticles decreased dramatically, closed to 0. The reason was that the isoelectric point of magnetic Co_0.5_Mn_0.5_Fe_2_O_4_ nanoparticles was 8. The surface of magnetic Co_0.5_Mn_0.5_Fe_2_O_4_ nanoparticles had a positive charge when the pH of CR solution was less than 8, and it interacted electrostatically with CR. The positive charge on the surface of magnetic Co_0.5_Mn_0.5_Fe_2_O_4_ nanoparticles gradually decreased with an increase in pH of the CR solution, rsulting in a gradual decrease in adsorption capacity. The surface of magnetic Co_0.5_Mn_0.5_Fe_2_O_4_ nanoparticles had a negative charge when the pH of CR solution was greater than 8, and it existed a repulsive force with CR. In addition, under alkaline conditions, the competition between CR molecules and OH^-^ led to the decrease of adsorption capacity for magnetic Co_0.5_Mn_0.5_Fe_2_O_4_ nanoparticles.

### Stability and recyclability of Co_0.5_Mn_0.5_Fe_2_O_4_

**Fig 9:**
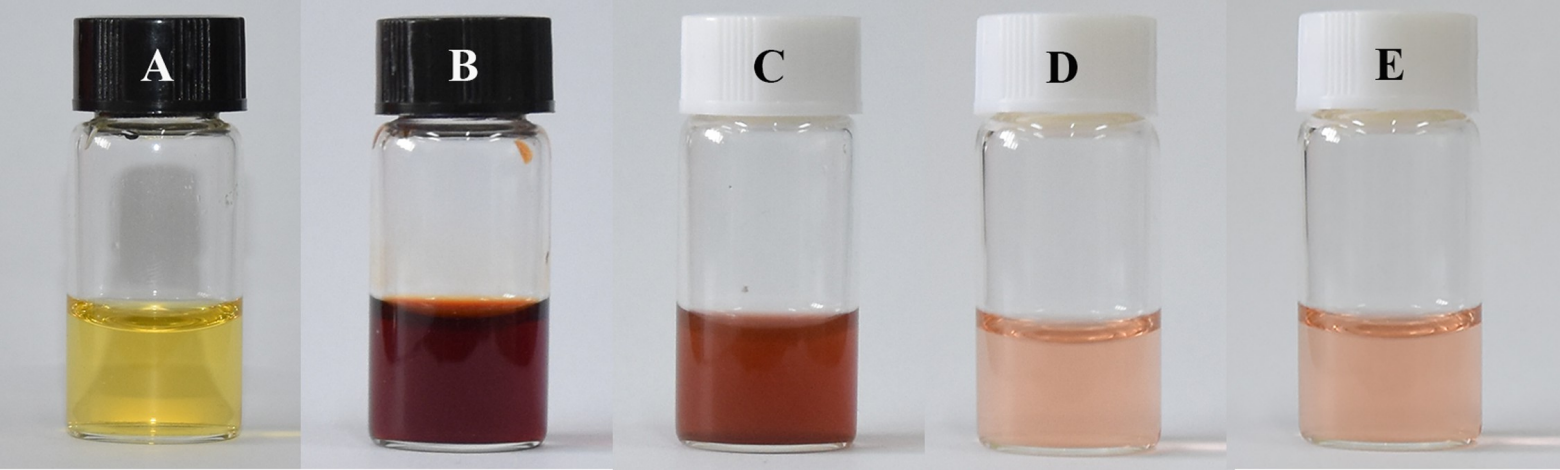
The photographs of Fe^3+^ solution (A), Fe^3+^ solution driped NaSCN (B), CR solution (C), CR solution after adsorption (D), and CR solution afetr adsorption and driped NaSCN (E).

In the adsorption process, the stability of adsorbent was of greater importance, which could be explored by whether the material decomposes other ions. To explore whether Fe^3+^ and NaSCN reacted, the color changed from yellow to blood red (Fig 9B) when 3 drips of NaSCN solution were added into the Fe^3+^ solution (Fig 9A), the reason was that Fe^3+^ reacted with SCN^-^ to form Fe(SCN)_3_. Comparing CR solution (Fig 9C) with adsorbed CR solution (Fig 9D), it could be seen that the color degree of CR decreased significantly after adsorption. After adding 3 drops of NaSCN solution, there was no change in the supernatant color of CR adsorbed onto magnetic Co_0.5_Mn_0.5_Fe_2_O_4_ nanoparticles (Fig 9E), compared to Fig 9B, indicating that Fe^3+^ was not released from magnetic Co_0.5_Mn_0.5_Fe_2_O_4_ nanoparticles in the process of CR adsorbed onto magnetic Co_0.5_Mn_0.5_Fe_2_O_4_ nanoparticles, which suggested that magnetic Co_0.5_Mn_0.5_Fe_2_O_4_ nanoparticles were stable in the CR solutions.

**Fig 10:**
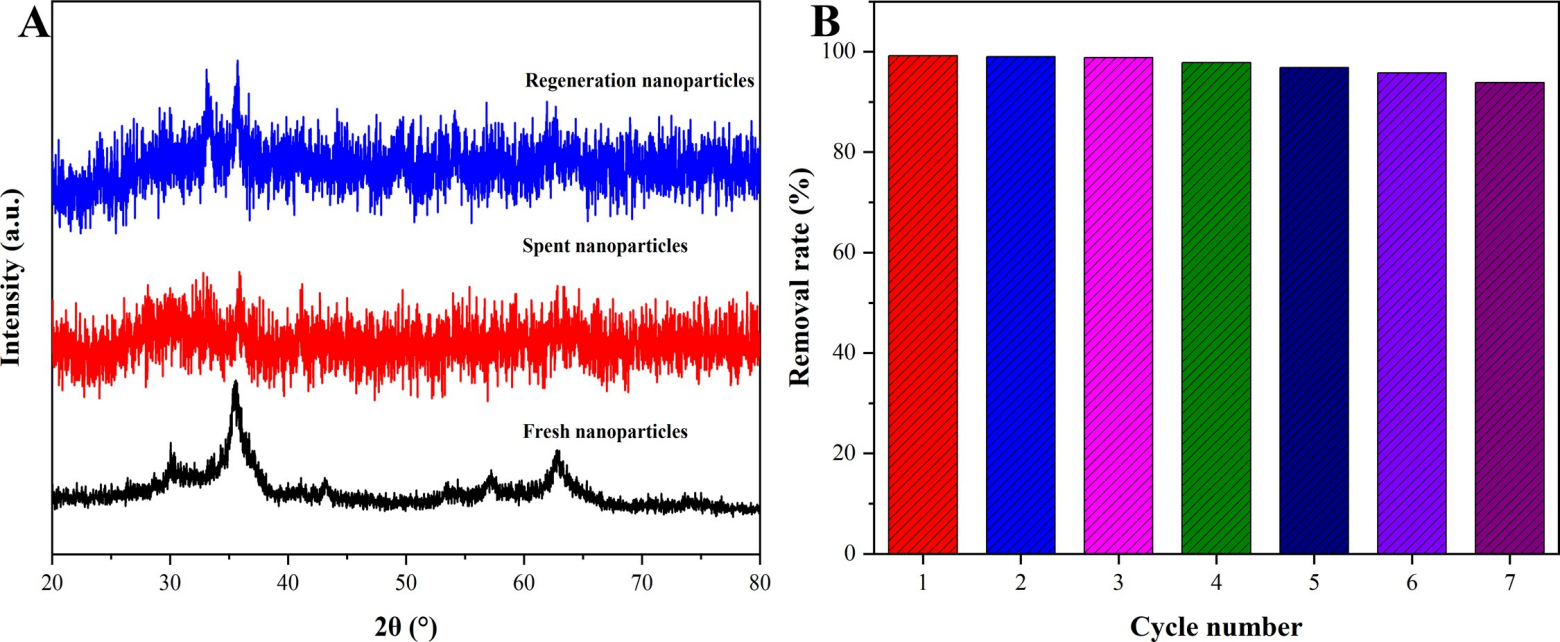
The XRD patterns of fresh nanoparticles, spent nanoparticles and regeneration nanoparticles (A) and the recycling of magnetic Co_0.5_Mn_0.5_Fe_2_O_4_ nanoparticles (B).

The XRD patterns of fresh nanoparticles, spent nanoparticles, and regeneration nanoparticles were shown in Fig 10A, it could be seen that the intensity of the diffraction peak decreased after CR adsorbed onto magnetic Co_0.5_Mn_0.5_Fe_2_O_4_ nanoparticles. The diffraction intensity of 32.9^o^ and 35.5^o^ increased after re-calcination because the removal of CR during the calcination process. This might be caused by the prolongation of calcination time, similar to the results of magnetic Co_0.5_Mn_0.5_Fe_2_O_4_ nanoparticles prepared by Ling et al [54]. The results indicated that the stability of magnetic Co_0.5_Mn_0.5_Fe_2_O_4_ nanoparticles was good. The high-cost performance of the adsorbents was more popular in practical applications [55]. Among many factors to evaluate the cost performance of adsorbent, the recycling numbers of adsorbent were particularly important. Fig 10B showed that the relative removal rate of CR adsorbed by magnetic Co_0.5_Mn_0.5_Fe_2_O_4_ nanoparticles could still reach 93.85% of the first time after 7 cycles (the corresponding raw data were listed in S5 Table). Compared to the removal rates reported in relevant studies, HPCs-4-700 studied by Wang et al. was only 78.66% after 5 cycles [51], MNC HS studied by Wang et al. was only 76.6% after 5 cycles [50], indicating that magnetic Co_0.5_Mn_0.5_Fe_2_O_4_ nanoparticles had a better recycling performance. The more the recycling number was, the lower the removal rate of magnetic Co_0.5_Mn_0.5_Fe_2_O_4_ nanoparticles was. The reason was that the specific surface area of magnetic Co_0.5_Mn_0.5_Fe_2_O_4_ nanoparticles might diminish due to repeated calcination. The decrease in adsorption capacity might be that the void collapse of magnetic Co_0.5_Mn_0.5_Fe_2_O_4_ nanoparticles led to the gradual reduction of adsorption sites in the process of recalcination [56].

### Electrochemical

**Fig 11:**
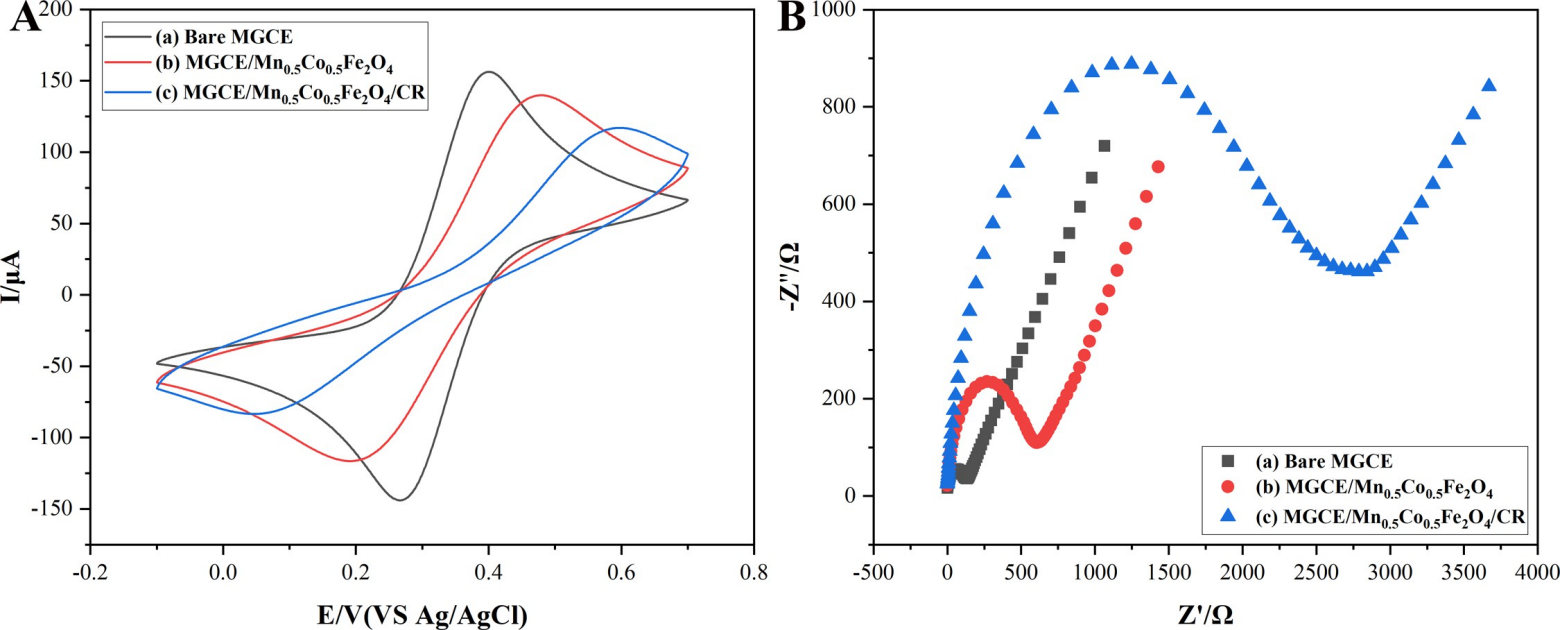
The CV curves (A) and EIS curves (B) of Bare MGCE (a), MGCE/Co_0.5_Mn_0.5_Fe_2_O_4_ (b), MGCE/Co_0.5_Mn_0.5_Fe_2_O_4_ /CR (c).

Electrochemical experiments were conducted to study the changes of current and resistance caused by the adsorbed CR onto magnetic Co_0.5_Mn_0.5_Fe_2_O_4_ nanoparticles. The CV curve of CR adsorbed onto magnetic Co_0.5_Mn_0.5_Fe_2_O_4_ nanoparticles was displayed in Fig 11A. The current value decreased when magnetic Co_0.5_Mn_0.5_Fe_2_O_4_ nanoparticles were added to the bare electrode, the reason was that magnetic Co_0.5_Mn_0.5_Fe_2_O_4_ nanoparticles blocked the flow of [Fe(CN)_6_]^3-/4-^ to the electrode surface. The current value decreased further when CR was adsorbed onto magnetic Co_0.5_Mn_0.5_Fe_2_O_4_ nanoparticles due to an interaction between CR and magnetic Co_0.5_Mn_0.5_Fe_2_O_4_ nanoparticles, which prevented [Fe(CN)_6_]^3-/4-^ from reaching the current surface.

The EIS measurement results were represented by Nyquist plots. The diameter of the semicircle was related to the resistance of the material. The larger the diameter of the semicircle, the greater the resistance. Fig 11B revealed that the resistance value increased when magnetic Co_0.5_Mn_0.5_Fe_2_O_4_ nanoparticles were added to the bare electrode. The resistance value increased further when CR was adsorbed onto magnetic Co_0.5_Mn_0.5_Fe_2_O_4_ nanoparticles. The results indicated that CR was adsorbed onto magnetic Co_0.5_Mn_0.5_Fe_2_O_4_ nanoparticles, and the electrical conductivity of the magnetic Co_0.5_Mn_0.5_Fe_2_O_4_ nanoparticles decreased after the adsorption of CR. The decrease in electrical conductivity might be due to the poor electrical conductivity of CR.

## Conclusions

1. Magnetic Co_0.5_Mn_0.5_Fe_2_O_4_ nanoparticles were prepared via the combustion and calcination process, the average particle diameter of Co_0.5_Mn_0.5_Fe_2_O_4_ nanoparticles calcined at 400°C for 2 h with 20 mL absolute ethanol as the solvent and incendiary agent was 31.5 nm, and their saturation magnetization was 25.25 emu·g^-1^.
2. The adsorption of CR onto magnetic Co_0.5_Mn_0.5_Fe_2_O_4_ nanoparticles was consistent with the pseudo-second-order kinetic model and Freundlich isotherm model, indicating that CR adsorbed onto magnetic Co_0.5_Mn_0.5_Fe_2_O_4_ nanoparticles was a multilayer chemisorption process; the adsorption thermodynamics showed that the adsorption was a favored process.
3. The ionic strength of Cl^-^ in CR solution had no obvious effect on adsorption efficiency of magnetic Co_0.5_Mn_0.5_Fe_2_O_4_ nanoparticles, and the maximum adsorption capacity of CR onto magnetic Co_0.5_Mn_0.5_Fe_2_O_4_ nanoparticles was 58.3 mg·g^-1^ when the pH of CR solution was 2.
4. The ion leaching experiment and XRD proved that magnetic Co_0.5_Mn_0.5_Fe_2_O_4_ nanoparticles had good stability, and the relative removal rate of magnetic Co_0.5_Mn_0.5_Fe_2_O_4_ nanoparticles for the removal of CR could still reach 93.85% of the first time after 7 cycles, indicating that magnetic Co_0.5_Mn_0.5_Fe_2_O_4_ nanoparticles had a good application prospect.

In summary, magnetic Co_0.5_Mn_0.5_Fe_2_O_4_ nanoparticles were applied as a novel type of nanomaterial for the adsorption of CR in industrial wastewater. The simple preparation method, inexpensive and readily available raw materials made Co_0.5_Mn_0.5_Fe_2_O_4_ to be an excellent adsorption nanomaterial, providing a new development nanomaterial for industrial wastewater treatment. However, since the present researches were carried out under laboratory conditions, further studies on the device parameters and the practical influences involved are needed if it is to be used for large-scale practical applications.

## Supporting information

S1 Table Raw data for adsorption kinetics at different initial CR concentrations. (DOCX)

S2 Table The raw data for the adsorption isotherms of CR onto magnetic Co_0.5_Mn_0.5_Fe_2_O_4_ nanoparticles at 303 K (A), 313 K (B), and 323 K (C).
(DOCX)

S3 Table Raw data for effect of ion strength on the equilibrium adsorption capacity of CR onto magnetic Co_0.5_Mn_0.5_Fe_2_O_4_ nanoparticles. (DOCX)

S4 Table Raw data for effect of pH on the equilibrium adsorption capacity of CR onto magnetic Co_0.5_Mn_0.5_Fe_2_O_4_ nanoparticles. (DOCX)

S5 Table The raw data of Removal rate for the magnetic Co_0.5_Mn_0.5_Fe_2_O_4_ nanoparticles. (DOCX)
