# Supplementary material for: Adsorption of Congo red on magnetic cobalt-manganese ferrite nanoparticles: Adsorption kinetic, isotherm, thermodynamics, and electrochemistry
Source: PLoS One. 2024 Oct 9;19(10):e0307055. doi: 10.1371/journal.pone.0307055 (PMC11463770; doi:10.1371/journal.pone.0307055)
Supplement: S1 Table — (DOCX) [file pone.0307055.s001.docx]

**Table S1. Raw data for adsorption kinetics at different initial CR concentrations.**

| **Tine (min)** | **Initial concentration of CR (mg·L^-1^)** | | | |
| --- | --- | --- | --- | --- |
|  | **50** | **100** | **150** | **200** |
| 10 | 17.8012 | 29.3789 | 42.0248 | 38.0993 |
| 20 | 18.6708 | 34.0919 | 50.1988 | 51.2422 |
| 30 | 19.2029 | 35.7143 | 51.4907 | 56.8385 |
| 40 | 19.4252 | 37.0547 | 53.3416 | 61.2981 |
| 50 | 19.6385 | 37.5031 | 54.0932 | 63.0435 |
| 60 | 19.7385 | 37.7280 | 54.9665 | 66.0994 |
| 80 | 19.8285 | 38.3342 | 54.9441 | 66.7453 |
| 100 | 19.8630 | 38.4224 | 55.7640 | 66.9441 |
| 120 | 19.8588 | 38.8696 | 55.9130 | 67.9933 |
| 140 | 19.9130 | 38.9441 | 56.1615 | 68.7081 |
| 160 | 19.8740 | 39.0683 | 56.5839 | 68.1615 |
| 180 | 19.9130 | 39.0932 | 56.6335 | 70.1739 |
