# Supplementary material for: Adsorption of Congo red on magnetic cobalt-manganese ferrite nanoparticles: Adsorption kinetic, isotherm, thermodynamics, and electrochemistry
Source: PLoS One. 2024 Oct 9;19(10):e0307055. doi: 10.1371/journal.pone.0307055 (PMC11463770; doi:10.1371/journal.pone.0307055)
Supplement: S2 Table — The raw data for the adsorption isotherms of CR onto magnetic Co0.5Mn0.5Fe2O4 nanoparticles at 303 K (A), 313 K (B), and 323 K (C). (DOCX) [file pone.0307055.s002.docx]

**Table S2. The raw data for the adsorption isotherms of CR onto magnetic Co_0.5_Mn_0.5_Fe_2_O_4_ nanoparticles at 303 K (A), 313 K (B), and 323 K (C).**

| **303 K** | | **313 K** | | **323 K** | |
| --- | --- | --- | --- | --- | --- |
| **C_e_ (mg/L)** | **q_e_ (mg/g)** | **C_e_ (mg/L)** | **q_e_ (mg/g)** | **C_e_ (mg/L)** | **q_e_ (mg/g)** |
| 0.22 | 19.9130 | 0.28 | 19.8882 | 0.50 | 19.7988 |
| 2.27 | 39.0932 | 5.68 | 37.7267 | 10.84 | 35.6646 |
| 8.42 | 56.6335 | 17.42 | 53.0311 | 23.89 | 50.4472 |
| 24.57 | 70.1739 | 37.48 | 65.0062 | 43.89 | 62.4472 |
| 52.80 | 78.8820 | 67.27 | 73.0932 | 77.70 | 68.9193 |
| 77.39 | 89.0435 | 97.27 | 81.0932 | 109.69 | 76.1242 |
| 116.65 | 93.3416 | 134.53 | 86.1863 | 143.23 | 82.7081 |
