# Supplementary material for: Adsorption of Congo red on magnetic cobalt-manganese ferrite nanoparticles: Adsorption kinetic, isotherm, thermodynamics, and electrochemistry
Source: PLoS One. 2024 Oct 9;19(10):e0307055. doi: 10.1371/journal.pone.0307055 (PMC11463770; doi:10.1371/journal.pone.0307055)
Supplement: S3 Table — (DOCX) [file pone.0307055.s003.docx]

**Table S3. Raw data for effect of ion strength on the equilibrium adsorption capacity of CR onto magnetic** **Co_0.5_Mn_0.5_Fe_2_O_4_ nanoparticles.**

| **NaCl (M)** | 0 | 0.01 | 0.02 | 0.04 | 0.08 | 0.16 |
| --- | --- | --- | --- | --- | --- | --- |
| **Removel rate (%)** | 99.193 | 99.866 | 99.947 | 99.953 | 99.596 | 99.829 |
