# Supplementary material for: Adsorption of Congo red on magnetic cobalt-manganese ferrite nanoparticles: Adsorption kinetic, isotherm, thermodynamics, and electrochemistry
Source: PLoS One. 2024 Oct 9;19(10):e0307055. doi: 10.1371/journal.pone.0307055 (PMC11463770; doi:10.1371/journal.pone.0307055)
Supplement: S4 Table — (DOCX) [file pone.0307055.s004.docx]

**Table S4. Raw data for effect of pH** **on the equilibrium adsorption capacity of CR onto magnetic** **Co_0.5_Mn_0.5_Fe_2_O_4_ nanoparticles.**

| **pH** | 2 | 4 | 6 | 8 | 10 | 12 |
| --- | --- | --- | --- | --- | --- | --- |
| **q_e_ (mg/g)** | 58.2981 | 57.6522 | 55.6646 | 53.3789 | 2.4845 | 0 |
