# Supplementary material for: Adsorption of Congo red on magnetic cobalt-manganese ferrite nanoparticles: Adsorption kinetic, isotherm, thermodynamics, and electrochemistry
Source: PLoS One. 2024 Oct 9;19(10):e0307055. doi: 10.1371/journal.pone.0307055 (PMC11463770; doi:10.1371/journal.pone.0307055)
Supplement: S5 Table — (DOCX) [file pone.0307055.s005.docx]

**Table S5. The raw data of Removal rate for the magnetic Co_0.5_Mn_0.5_Fe_2_O_4_ nanoparticles.**

| **Cycle number** | 1 | 2 | 3 | 4 | 5 | 6 | 7 |
| --- | --- | --- | --- | --- | --- | --- | --- |
| **Removal rate (%)** | 99.193 | 99.027 | 98.820 | 97.826 | 96.832 | 95.797 | 93.851 |
